# Heparin-Based Biomaterials for Sustained Release of Growth Factors for Bone Tissue Engineering and Regeneration

**DOI:** 10.3390/jfb17030156

**Published:** 2026-03-22

**Authors:** Keisuke Nakayama, Xueqin Gao, Britney S. Force, Marc J. Philippon, Johnny Huard

**Affiliations:** 1 Linda and Mitch Hart Center for Regenerative and Personalized Medicine, Steadman Philippon Research Institute, Vail, CO 81657, USA; 2 The Steadman Clinic, Vail, CO 81657, USA

**Keywords:** heparin, bone tissue engineering, heparin/poly(ethylene argininylaspartate diglyceride) (PEAD) coacervate, heparin modified hydrogel, bone morphogenetic proteins, heparin modified nanoparticle, heparin modified microsphere

## Abstract

Large bone defects resulting from trauma, tumor resection, infection, or degenerative diseases pose a major clinical challenge in orthopedic surgery and regenerative medicine. Despite advances in biomaterials and surgical techniques, successful outcomes are often compromised by poor vascularization, limited osteoinduction, and donor-site morbidity associated with autografts or allografts. However, conventional delivery systems suffer from burst release, rapid clearance, off-target effects, and supraphysiologic dosing, which can lead to undesirable complications such as ectopic ossification and inflammation, with some reports raising concerns about the long-term tumorigenic risk. Heparin, a naturally highly sulfated glycosaminoglycan structurally related to heparan sulfate, has emerged as a particularly attractive candidate for affinity-based biomaterial systems. It naturally binds over 300 growth factors, including bone morphogenetic proteins. By protecting these proteins from enzymatic degradation, enhancing their bioavailability, and mediating receptor clustering, heparin provides both biochemical stability and biofunctional modulation. This review provides a comprehensive overview of heparin-based delivery strategies in bone tissue engineering. We begin by describing the biological functions of heparin in modulating growth factor activity. We then discuss in detail the different heparin-based biomaterials designed to sustain the release of growth factors for bone tissue engineering, including the heparin–polycation coacervate system; heparin-based supramolecules; and heparin-based hydrogels, nanoparticles, and microspheres for sustained release of bone morphogenic proteins and other growth factors for bone tissue engineering. Finally, we assess the clinical and translational relevance of heparin-based systems, identify key challenges, and outline future perspectives, highlighting the potential of these biomaterials for providing safer and more effective therapies for bone regeneration.

## 1. Introduction

Bone fractures typically heal through coordinated biological processes that include hematoma formation, callus development, and remodeling [1,2,3]. However, critical-sized defects resulting from trauma, tumor resection, infection, or congenital anomalies often exceed the body’s innate regenerative ability. Furthermore, the high prevalence of osteoporosis in aging populations worldwide has led to a significant increase in bone fractures and nonunion. These injuries are associated with prolonged morbidity, functional impairment, and substantial socioeconomic burden. Current clinical solutions include autologous bone grafts, which remain the ‘gold standard’ due to their osteogenic, osteoinductive, and osteoconductive properties, and allografts, which provide structural support but are compromised by immune rejection and limited biological activity. Both approaches suffer from significant drawbacks: autografts require invasive harvest with risk of donor-site pain and infection, while allografts carry the risk of disease transmission and inferior remodeling capacity [4]. The discovery and recombinant production of bone morphogenetic proteins (BMPs) revolutionized the field of bone repair. rhBMP-2 and rhBMP-7 have been used clinically in spinal fusion and nonunion fractures [5]. Their introduction initially generated optimism, as clinical trials reported enhanced fusion rates and reduced healing time compared to grafting alone. However, post-marketing studies revealed significant complications, particularly when rhBMP-2 was delivered at high doses using collagen sponges as carriers. Documented adverse effects include ectopic ossification, inflammatory reactions, osteolysis, and radiculitis, with some reports raising concerns about long-term tumorigenic risk [6]. Meta-analyses have shown that BMPs can indeed improve union rates; however, the clinical benefits must be balanced with the risks associated with supraphysiologic dosing and uncontrolled release, such as heterotopic bone formation [7].

Therefore, the development of novel strategies using sustained release of relatively lower doses of bone growth factors to treat segmental bone defects without incurring heterotopic bone formation or inflammation is needed. Many different biomaterials have been developed to sustain the release of growth factors for bone regeneration. Among these, the natural extracellular matrix heparin has been extensively studied for promoting bone defect healing or regeneration in preclinical studies. A recent review by Wang and colleagues summarized heparin-based biomaterials for regeneration of various tissues, with limited focus on bone tissue engineering [8]. Therefore, the purpose of this review is to focus on the application of heparin as a natural biomaterial for the delivery and sustained release of bone growth factors for bone repair.

## 2. Molecular Characteristics of Heparin

**Figure 1:**
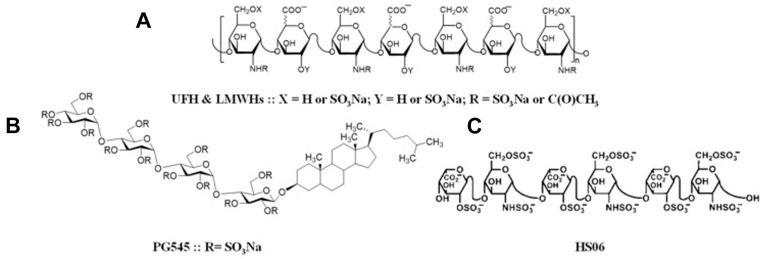
Heparin molecular structure. (**A**) Unfractionated or lower-molecular-weight heparin. (**B**) Synthetic heparin sulfate mimetic pixatimod (PG545). (**C**) Heparin sulfate S06. (Cited from Gatica Portillo D.R et al. [9].)

Heparin is a linear, highly sulfated glycosaminoglycan composed of repeating disaccharide units. Heparin’s molecular structure is shown in Figure 1, as reported by Gatica Portillo D.R et al. [9].

It is best known for its anticoagulant properties, but beyond hemostasis, heparin and its structural relative, heparan sulfate, bind growth factors via electrostatic and sequence-specific motifs. Natural heparin can bind to over 300 extracellular proteins, including many growth factors [10,11]. These interactions protect proteins from degradation, enhance bioavailability, and allow for the formation of gradients that direct cell migration and differentiation [12]. At the molecular level, glycosaminoglycan–protein interactions are predominantly governed by electrostatic attraction between negatively charged sulfate and carboxyl groups of heparin and clusters of positively charged basic amino acid residues—particularly lysine and arginine—within heparin-binding domains of target proteins [13]. These domains often contain concentrated basic residue arrangements that facilitate multivalent interactions, thereby increasing binding stability and avidity. Structural investigations using nuclear magnetic resonance (NMR), molecular modeling, and glycosaminoglycan microarray technologies have further clarified how three-dimensional conformation, charge distribution, and domain organization collectively influence binding affinity and selectivity. Importantly, interaction specificity is not determined solely by overall charge density. Variations in sulfation pattern, chain length, and domain distribution along the glycosaminoglycan backbone generate spatially organized interaction sites that modulate protein recognition and downstream signaling events. Such structural heterogeneity produces regulatory complexity beyond simple electrostatic attraction, underscoring the central role of structure–function relationships in heparin and heparan sulfate biology. Systems-level analyses have demonstrated that heparin- and heparan sulfate-binding proteins form highly interconnected molecular networks enriched in signaling, developmental, and regulatory pathways [14]. Further analyses suggest that glycosaminoglycan-binding proteins may function as coordinated signaling hubs rather than isolated binding partners, emphasizing the broader biological significance of these molecular interactions. In the context of bone regeneration, these physicochemical properties are particularly advantageous, as they enable stabilization and controlled release of osteoinductive factors such as BMPs and angiogenic mediators including vascular endothelial growth factor (VEGF). By extending protein half-life and modulating signaling intensity, heparin-based carriers enhance the therapeutic potential of growth factor delivery [15].

## 3. Natural Biological Functions of Heparin

Naturally, heparin is synthesized predominantly by connective tissue-type mast cells, where it is produced through the heparan sulfate biosynthetic pathway but undergoes more extensive sulfation. Newly synthesized heparin chains are attached to the serglycin core protein and stored within mast cells [16].

Heparin plays diverse biological roles beyond its well-known anticoagulant activity, mediated through binding to antithrombin III. Heparin and its structural analog heparan sulfate are critical modulators of cell signaling in tissue development and repair. Their ability to bind, protect, and present growth factors to their receptors makes them indispensable in regulating processes such as angiogenesis, osteogenesis, and chondrogenesis [17]. Through these interactions with many functional proteins, heparin regulates the availability and presentation of multiple growth factors, including BMPs, fibroblast growth factors (FGFs), VEGF, platelet-derived growth factor (PDGF), and transforming growth factor beta (TGF-β). By stabilizing these proteins and storing them within the extracellular matrix, heparin helps regulate the local distribution of signaling molecules involved in angiogenesis, osteogenesis, and tissue repair [18,19]. Previous studies have demonstrated that human BMP-2 contains a specific heparin-binding site; mutation of this site significantly reduces its biological activity [20]. Structural analyses have further revealed that variations in heparin sulfation patterns directly influence binding affinity and downstream signaling outcomes [13].

Beyond simple protection, heparin enhances receptor-mediated signaling. For FGFs, binding to heparin is an absolute requirement for receptor dimerization and activation; without heparin, FGF fails to effectively activate downstream signaling [21]. Similar mechanisms have been described for VEGF and BMPs, where heparin not only prolongs protein half-life but also enhances ligand–receptor interactions by facilitating co-receptor clustering at the cell surface [13]. Heparan sulfate proteoglycans (HSPGs), which are cell surface- or extracellular matrix (ECM)-bound relatives of heparin, also play critical roles in bone biology. HSPGs regulate VEGF gradients during skeletal repair and coordinate BMP signaling during endochondral ossification [19]. Knockout models deficient in specific HSPG core proteins exhibit impaired bone growth and angiogenesis, highlighting the physiological importance of glycosaminoglycan–growth factor interactions. Recent advances include the design of heparin-mimetic peptides and polymers that reproduce the sulfated domains required for growth factor binding. Laminin- and fibronectin-derived heparin-binding motifs, when incorporated into hydrogels, improve retention of BMPs and VEGF and enhance osteogenic and angiogenic repair [19,22].

One of the limitations of clinical translation is the anticoagulant activity of heparin. However, when heparin is incorporated into biomaterial scaffolds, interactions with growth factors and matrix components allow for localized growth factor release and the risk of systemic anti-coagulation is minimized [22,23]. There are several studies using heparin biomaterials with fibrin sealant without reporting any excessive bleeding at local heart, bone, or cartilage defects [24,25,26]. Further, there is non-coagulant heparin available [27]. For example, 2-O,3-O-desulfated heparin (ODSH) retains protein-binding affinity while exhibiting reduced interaction with clotting factors. Preclinical studies further suggest that these derivatives retain many of the biological activities of heparin while exhibiting markedly reduced anticoagulant activity, thereby minimizing the risk of hemorrhagic complications [28]. Therefore, the risk of a systemic anticoagulant effect is very minimal when using heparin-based biomaterials.

Taken together, the biological functions of heparin extend far beyond anticoagulation. Heparin acts as a multifunctional regulator of growth factor biology: it binds and stabilizes key osteogenic and angiogenic proteins, protects them from enzymatic degradation, modulates receptor clustering, and enables spatially organized presentation [13,18]. Engineering advances, including low-anticoagulant derivatives and heparin-mimetic peptides, further expand their translational potential.

## 4. Heparin–Polycation Coacervate Sustained Release System for Bone Tissue Engineering

Over the past two decades, heparin has been incorporated into a wide variety of biomaterial platforms. Early approaches included surface coatings and simple hydrogels, which improved factor retention but suffered from burst release. More advanced systems now exploit electrostatic self-assembly, chemical conjugation, and microfabrication to achieve controlled and sustained release of growth factors. Notably, the heparin–polycation coacervate system developed by Wang and colleagues represents a milestone in this evolution and is considered one of the most influential advancements [29]. Numerous studies have since applied heparin-based coacervates, hydrogels, scaffolds, and nanoparticles to deliver BMPs, FGFs, VEGF, and other molecules in preclinical models. This system exploits the electrostatic complexation between poly(ethylene argininylaspartate diglyceride) (PEAD), a synthetic polycation, and heparin to generate a stable liquid–liquid phase separated material known as a coacervate [30,31]. The resulting microdroplets encapsulate heparin-binding proteins at high efficiency, preserve their structural integrity, and shield them from proteolytic degradation while enabling sustained release.

### 4.1. Heparin–PEAD Coacervate for Sustained Release of Single Growth Factor for Bone Tissue Engineering

Heparin–PEAD coacervate was first developed for the delivery of FGF-2 to promote angiogenesis, which is also very important for bone regeneration. The results demonstrated FGF-2 bound by heparin formed a coacervate with PEAD through electrostatic forces, retained FGF-2 bioactivity, and significantly enhanced angiogenesis compared to bolus delivery of FGF-2 in a card model [29]. Subsequent studies showed that nerve growth factor (NGF) and VEGF could also be delivered effectively using the heparin–PEAD coacervate, resulting in enhanced neurotrophic and angiogenic signaling in vivo [32]. These studies proved the effectiveness of the heparin–PEAD coacervate for the sustained release of different growth factors.

With the success of the heparin–PEAD coacervate in the sustained release of FGF-2, NGF, and VEGF, it was subsequently used for the delivery of BMP-2 to promote bone regeneration. Li H et al. reported that BMP-2 encapsulated within the heparin–polycation coacervate promoted robust osteogenic differentiation of muscle-derived stem cells in vitro and enhanced bone formation in a muscle pocket ectopic bone formation model in mouse thigh muscle [33]. Further, heparin–PEAD coacervate-loaded BMP-2 has been shown to enhance osteogenic differentiation of human mesenchymal stem cells (MSCs) via increasing alkaline phosphate activity. Heparin–PEAD coacervate with BMP-2 incorporated into thiolated gelatin/poly(ethylene glycol) diacrylate (PEGDA) interpenetrating (IPN) composite hydrogels was further validated to promote bone regeneration in a rat critical-sized calvarial bone defect model, as demonstrated by a significantly high score for bone bridging and union according to micro-CT and histology [34]. Heparin–PEAD coacervate can not only sustain BMP-2 release but can also sustain the release of other BMPs. Using a heparin–PEAD coacervate release platform, Gao X et al. evaluated five BMPs (BMP-2, BMP-4, BMP-6, BMP-7, and BMP-9) for their effects on promoting osteogenic differentiation of human MSCs in vitro and bone regeneration in vivo using a mouse critical-sized calvarial bone defect model. They revealed that all five BMPs were functional when released in a sustained manner with heparin–coacervate in vitro and could promote bone regeneration in a critical-sized calvarial bone defect model using a fibrin sealant scaffold. BMP-2 and BMP-7 were identified as the most potent BMPs to promote bone defect healing without inducing heterotopic bone formation and inflammation. The quality of the newly regenerated bone formed by all BMPs delivered via coacervate was equivalent to that of the host bone, consisting of the bone matrix and bone marrow with normal bone architecture. This study also demonstrated that heparin–PEAD coacervate did not affect local coagulation, even when used simultaneously with a fibrin sealant, and provides evidence for its safety [25]. These studies demonstrate that heparin can bind and release many BMPs in a sustained manner for promoting osteogenic differentiation of stem cells and enhanced bone repair but does not affect hemostasis.

Interestingly, recent evidence has revealed that the differential osteogenic potency among BMP isoforms is closely linked to their intrinsic heparin-binding capability. Siverino et al. reported that although BMP-9 exhibits the strongest osteogenic activity in vitro, it shows limited bone formation in vivo, largely because it lacks heparin-binding motifs present in other osteogenic BMPs such as BMP-2. To address this limitation, the authors engineered a BMP-9 variant incorporating BMP-2-derived heparin-binding sequences (BMP-9 HB), which markedly increased its heparin affinity and ECM retention. When adsorbed onto collagen scaffolds and implanted subcutaneously in rats, BMP-9 HB induced significantly higher bone volume and density than either native BMP-2 or BMP-9, even at tenfold lower doses at 6 weeks after implantation. These findings demonstrate that effective heparin binding is a key determinant of BMP osteogenic efficacy, and that molecular engineering of heparin-binding domains represents a promising strategy for enhancing the translational potential of BMP-based regenerative therapies [35].

### 4.2. Heparin–PEAD Coacervate for Dual Delivery of Protein Factors for Tissue Engineering

Beyond single-factor delivery, the coacervate platform has been adapted for dual- or multi-factor release. Patel et al. demonstrated that simultaneous delivery of VEGF and BMP-2 synergistically enhanced bone regeneration in rat calvarial defect models relative to either factor alone [36]. Simultaneous delivery of BMP-2 and sFLT1 synergistically improved MIA-induced osteoarthritis, with similar effects to lent-BMP-2 transduced human muscle-derived stem cells [37]. Dual delivery of IGF1 and TGFβ3 with heparin–PEAD coacervate also enhanced neovascularization in a mouse skin flap model [38]. Heparin–PEAD coacervate-based dual delivery of TGFβ3 and interleukin 10 (IL10) promoted scarless skin regeneration in a rat skin wound defect model [39]. Controlled dual delivery of FGF-2 and IL10 significantly enhanced infarcted cardiac repair [40]. These results demonstrate that heparin–PEAD coacervate can not only bind and release single growth factors in a sustained manner but can also be used for delivering multiple factors which mimic natural healing processes that often involve multiple factors that act on different tissue cells.

### 4.3. Heparin-Based Sustained Release of Growth Factors for Repair of Other Musculoskeletal Tissues

The modularity of the coacervate extends further to other musculoskeletal tissues. IGF-1 delivered via a heparin–polycation coacervate in combination with adipose stem cells significantly improved cartilage matrix deposition in a rabbit full-thickness osteochondral defect model [41]. More recently, it was demonstrated that heparin–PEAD could release BMP-2,4,6,7,9 in a sustained manner and promote microfracture-mediated cartilage repair in a rat model by enhancing subchondral bone healing and up-regulation of SOX-9 expression. Heparin–PEAD coacervate-mediated sustained release of BMP-2,4,6,7,9 did not incur any heterotopic bone formation in the injured site [26]. These applications highlight the potential of coacervates not only for bone but also for cartilage regeneration. It is also indicated that the heparin in the coacervate does not affect hemostasis even in the environment of a microfracture specifically used to induce subchondral bone bleeding to release host stem cells from bone marrow.

Collectively, heparin–polycation coacervates represent a milestone in controlled growth factor delivery. Their ability to release a wide range of heparin-binding proteins in a sustained manner, preserve bioactivity, reduce required doses, and enable both single and combinatorial delivery modes makes them a highly promising strategy for translational bone regeneration [29,32].

## 5. Heparin-Based Supramolecular Nanostructure for Delivery of Growth Factors for Bone Regeneration

Stupp SI’s group also took advantage of heparin sulphate (HS)’s natural capacity to bind BMP-2 and designed biomimetic heparin-binding peptide amphiphile (HBPA) nanofibers that can bind heparin sulfate to form a supramolecular gel that can release BMP-2 in a sustained manner. This supramolecular gel can release BMP-2 for longer than the PA nanofiber gel without HS in vitro. Furthermore, incorporation of this supramolecular gel loaded with 1 μg BMP-2 in a collagen sponge and implanted into 5 mm rat critical-sized long bone defects resulted in significantly higher bone bridge rates and the highest bone volumes in the COL+HBPA+HS+BMP-2 group than in groups without HS or PA nanofibers, as demonstrated by micro-CT. Histology also showed more mature bone in this group [42]. This research group also designed supramolecular sulfated glycopeptide nanostructures with a trisulfated monosaccharide on their surfaces that can bind five critical proteins with different polysaccharide-binding domains. The glycopeptide nanostructures amplified signaling of BMP-2 significantly more than the natural sulfated polysaccharide heparin, and promoted the regeneration of bone in a rat spine fusion model with a protein dose 100-fold lower than that required in the animal model [43]. More recently, McClendon et al. extended these supramolecular design principles to develop a supramolecular polymer–collagen microparticle slurry that combines a heparin-mimetic polymer with porous collagen particles, forming a paste-like injectable gel that stiffens in situ. This biomaterial achieved complete spinal fusion in rabbits with only 5 μg BMP-2 per implant—over 100-fold lower than current clinical doses—while maintaining robust bone regeneration. This study further supports the concept that precise molecular organization and affinity-guided growth factor presentation can greatly enhance osteogenic efficacy even at ultralow protein doses [44]. Interestingly, Newcomb et al. further revealed that the nanoscale cohesion and hydrogen bonding strength of peptide-based supramolecular nanofibers can directly modulate intracellular signaling rather than merely release kinetics. They found that weakly cohesive peptide nanostructures, when combined with low concentrations of BMP-2 or Wnt, markedly enhanced osteogenic and myogenic signaling by increasing membrane lipid raft mobility, leading to more efficient receptor clustering and downstream SMAD activation. In contrast, nanostructures with stronger internal hydrogen bonding reduced growth factor signaling. This work introduced the new concept that dynamic supramolecular organization can potentiate growth factor pathways at the cell–material interface, providing mechanistic insight into the high bioactivity observed in subsequent heparin-based supramolecular systems [45]. These studies demonstrated that adding heparin or a heparin-binding domain analogue molecular structure to the supramolecules could significantly reduce the dose of BMP-2 and enhance BMP-2 activity and bone regeneration efficiency and reduce unnecessary side effects.

## 6. Heparin-Modified Hydrogels and Scaffolds for Bone Tissue Engineering

Beyond heparin–PEAD coacervate and HBPA–heparin supramolecular systems for the sustained release of bone growth factors, heparin-functionalized hydrogels and scaffolds have been explored as delivery platforms for bone tissue engineering. These systems combine structural support with affinity-based binding of growth factors, allowing for controlled and localized release that mimics the natural ECM.

### 6.1. Heparin Composite Hydrogel for Sustained Release of Growth Factors for Bone Tissue Engineering

Early studies developed PEG–heparin hydrogels capable of binding TGF-β1 and fibroblast growth factor-2, which demonstrated sustained release profiles and superior osteogenic activity compared to unmodified PEG carriers [46]. Fibrin–heparin composites were subsequently introduced and demonstrated markedly improved BMP-2 retention compared with collagen sponges, resulting in superior bone regeneration in critical-sized defect models [47]. These findings reinforced the concept that heparin conjugation enhances the therapeutic effects of growth factors by reducing burst release and prolonging bioactivity.

Heparin-modified hyaluronic acid hydrogels demonstrated sustained BMP-2 delivery and effective bone regeneration in vivo [48]. More recently, Zhou T et al. developed an injectable and self-healing carboxymethyl chitosan/polyethylene glycol/heparin sulfate (CMCS/PEG/HS) hydrogel designed for the controlled release of BMP-2. The negatively charged heparin sulfate component enhanced BMP-2 binding and prevented premature degradation, leading to degradation-dependent rather than diffusion-controlled release kinetics. This system exhibited high injectability, biocompatibility, and adaptability to irregular bone defects, achieving significant osteogenic effects in a rat skull defect model. These findings underscore the promise of heparin-functionalized injectable hydrogels as minimally invasive delivery platforms for bone regeneration [49]. Ma C et al. developed a hydrogel made of gelatin–heparin–tyramine for the sustained release of BMP-2 to treat juvenile idiopathic osteonecrosis of the femoral head (ONFH). In vitro studies showed that the gelatin–heparin–tyramine hydrogel retained BMP-2 for four weeks. The injection of the hydrogel efficiently prevented BMP2 leakage. When preceded by a bone wash technique, the injected hydrogel had a broad distribution in the femoral head. In vivo studies on pigs revealed that the bone wash procedure followed by administration of BMP-2-hydrogel produced homogeneous bone regeneration without HO. It preserved the subchondral contour and restored subchondral endochondral ossification. This study demonstrated a promising BMP-2-hydrogel treatment for ONFH treatment, especially for teenagers [50].

In addition to conventional PEG- and hyaluronic acid-based hydrogels, multifunctional heparin composites have recently been developed to simultaneously enhance biochemical activity, antioxidant capacity, and mechanical strength. Wu et al. engineered a gelatin/poly (ethylene glycol) diacrylate (GPEGD) hydrogel functionalized with polydopamine/heparin nanoparticles (BPDAH), which enabled BMP-2 loading, reactive oxygen species (ROS) scavenging, and increased stiffness. In a mandibular bone defect model, BPDAH–GPEGD hydrogels significantly promoted new bone formation and improved the quality of regenerated tissue, demonstrating that multiscale, ROS-responsive heparin-based hydrogels can address both biological and mechanical challenges in bone tissue engineering [51]. Advances in scaffold design have enabled the development of materials with tunable architectures, mechanical properties, and degradation kinetics that also enhance local growth factor retention. Hettiaratchi MH and colleagues further demonstrated that embedding heparin microparticles within collagen–hydroxyapatite scaffolds confined BMP-2 activity to the defect site, thereby reducing ectopic ossification and improving spatial specificity [52]. Extending these structural advances, Brown et al. developed a heparin-containing hydrogel/3D-printed scaffold composite for craniofacial reconstruction. This platform integrated hydrolytically degradable heparin-based hydrogels with 3D-printed porous scaffolds via a rapid mold-injection process, achieving homogeneous hydrogel distribution and sustained release of heparin-binding molecules for 14 days. The composites exhibited tunable viscoelastic stiffness comparable to trabecular bone or auricular cartilage while maintaining high cell viability, demonstrating the translational potential of heparin-based composite scaffolds that combine mechanical reinforcement with controlled biologic delivery [53]. More recently, Li X et al. developed a multifunctional nanofiber membrane carrying angiogenic, dentinogenic, and neurogenic growth factors for dental pulp regeneration. They used electrospun gelatin/polycaprolactone (GEL/PCL) nanofiber membranes modified with heparin (H-GEL/PCL) and loaded with VEGF, BMP-2, and NGF. It was found that the H-GEL/PCL membranes exhibited uniform nanofiber morphology, favorable mechanical and hydrophilic properties, sustained degradation, and controlled growth factor release. H-GEL/PCL membranes loaded with VEGF, BMP-2, and NGF enhanced angiogenic, odontogenic, and neurogenic differentiation of dental pulp stem cells in vitro and facilitated vascular and neural ingrowth of dental pulp-like tissue in vivo [54].

Additionally, functionalization of biomaterial matrices with laminin heparin-binding domains (HBDs) improves the retention of growth factors such as VEGF and PDGF-BB in fibrin matrices, significantly enhancing in vivo angiogenic outcomes [55]. Ku CY et al. designed a heparan sulfate proteoglycan 2(HSPG2)-coated poly (lactic acid) (PLA) scaffold to enhance FGF delivery and promote cranial bone regeneration. In vitro characterization showed that this scaffold has an ideal 0.3 mm pore size and 60% porosity, enabling MG63 cell proliferation and osteogenesis. The addition of HSPGs helped modulate FGF signaling during MG63 cell differentiation. In vivo, these novel 3D-printed PLA scaffolds coated with HSPG2 created an osteoconductive environment and repaired cranial bone defects by regulating FGF delivery via HSPG2/FGF signaling [56].

Furthermore, Ma L et al. developed a heparin-crosslinked fish-derived collagen scaffold to improve the stability and growth factor binding capacity of Nile tilapia collagen through EDC/NHS-mediated conjugation. The resulting heparin–collagen composite (HC-COL) exhibited enhanced thermal stability, superior BMP-2 affinity, and excellent cytocompatibility. In vitro, BMP-2-loaded HC-COL significantly increased alkaline phosphatase (ALP) activity and mineralized nodule formation in MC3T3-E1 cells, while in vivo implantation in rat calvarial defects resulted in greater mineralized and mature bone formation compared with unmodified collagen. This study highlights that heparin conjugation can not only improve biochemical functionality but also expand the applicability of non-mammalian collagen sources for bone tissue engineering [57]. The combination of stem cells with heparinized scaffolds has been another rapidly expanding area. For example, sulfated chitosan hydrogels combined with mesenchymal stem cells (MSCs) have been shown to enhance osteogenesis and vascularization in bone defect models [58].

In addition to improving factor retention, heparin-modified scaffolds can modulate the host immune response. Recent evidence suggests that heparin incorporation reduces macrophage-mediated inflammation and promotes a pro-regenerative phenotype, which may further contribute to enhanced healing [4].

Taken together, these findings indicate that heparin can be used with many different hydrogels or scaffolds, enhance growth factor retention and bioactivity, and allow for the delivery of multiple functional factors including osteogenic and angiogenic ones to promote bone regeneration locally without affecting systemic hemostasis.

### 6.2. Heparin Conjugated Inorganic Scaffold for Bone Tissue Engineering

Extending the application of heparin-based delivery to inorganic scaffolds, Tang et al. developed a 3D-printed calcium phosphate (CaP) ceramic integrated with heparin/polyethylenimine (PEI) nanogels loaded with BMP-2. The nanogels were immobilized onto the CaP surface through dopamine/dihydroxyphenylacetic acid (DA/DOPAC)-assisted adhesion, providing a biomimetic interface that combined a high BMP-2 loading efficiency with sustained local release. This composite scaffold promoted bone marrow stromal cell proliferation, osteogenic gene expression, and mineralized matrix deposition in vitro, and significantly enhanced bone regeneration in vivo compared with unmodified ceramics. These findings highlight how heparin-based nanogels can be synergistically combined with 3D-printed ceramics to improve biological performance, bridging the gap between bioactivity and mechanical strength in next-generation bone substitutes [59]. Chen et al. developed a heparinized gelatin–hydroxyapatite–tricalcium phosphate (HG–HA–TCP) scaffold loaded with sustained-release VEGF. This composite exhibited excellent biocompatibility and promoted both osteogenic differentiation and angiogenesis in vitro and in vivo, demonstrating that heparin modification enhances growth factor stability and coupling between vascular and bone regeneration processes [60]. Vater C et al. developed heparin-modified mineralized collagen scaffolds functionalized with naturally occurring bioactive factor mixtures (platelet concentrates, adipose tissue, and cell secretomes) and/or rhBMP-2. After implantation into a 2 mm segmental femoral defect in mice, micro-CT and histology demonstrated that the bioactive factor mixtures were inferior to the use of rhBMP-2 in terms of new BV and degree of defect healing when using this heparin-modified scaffold. Neither increasing the concentration of rhBMP-2 nor combining it with the bioactive factor mixtures led to a further enhancement of defect healing [61]. These studies demonstrated that heparin can also be incorporated into inorganic scaffolds to bind therapeutic proteins, release them in a sustained manner locally, and promote bone regeneration.

### 6.3. Heparin-Based Hydrogel for Engineering Other Musculoskeletal Tissues 

Sun X et al. reported a collagen/chitosan/0.5 silk fibroin composite scaffold incorporating polylysine–heparin sodium nanoparticles for TGF-β1 delivery (COL/CS/0.5SF-TPHNs). This system enabled controlled, sustained release of TGF-β1 and demonstrated favorable biocompatibility in vitro, supporting the adhesion and proliferation of mouse mesenchymal stem cells (mBMSCs). Furthermore, in vivo evaluation demonstrated enhanced cartilage regeneration and subchondral bone healing in a rabbit cartilage defect model [62]. Sarsenova et al. fabricated a heparin-conjugated fibrin (HCF) hydrogel co-loaded with synovium-derived mesenchymal stem cells (SDMSCs), transforming growth factor-β1 (TGF-β1), and bone morphogenetic protein-4 (BMP-4) for osteochondral defect repair. The HCF hydrogel demonstrated excellent biocompatibility, slowed degradation, and sustained release of both growth factors over 4 weeks. In a rabbit osteochondral defect model, combined delivery of SDMSCs with TGF-β1 and BMP-4 synergistically enhanced the regeneration of hyaline cartilage and subchondral bone compared with single-component treatments. This study highlights how heparin-modified hydrogels can serve as multiple regenerative factor carriers, enabling the coordinated delivery of stem cells and multiple signaling molecules to achieve both chondrogenic and osteogenic regeneration within complex osteochondral environments [63].

Lin C et al. designed a delivery system using heparin by targeting T helper 17 (Th17) cells, which exacerbate osteochondral tissue degradation via their pro-inflammatory cytokine interleukin-17 (IL-17) in the early stages of osteochondral defects (OCDs) heali, with anti-inflammatory factor IL-4 and the delivery of TGF-β1 to promote cartilage repair. Rapid IL-4 release from methacrylated hyaluronic acid (HAMA) hydrogel exerts a potent immunomodulatory effect by inhibiting the differentiation and function of Th17 cells while TGF-β1, anchored on methacrylated hyaluronic acid, and heparin (HAMA@HepMA) microparticles provide sustained regenerative signals. This approach synergistically converts the pro-inflammatory microenvironment into a pro-regenerative niche for enhanced OCD healing in a rat osteochondral defect model [64].

These studies indicate that heparin can bind diversified factors and sustain release for osteochondral defect cartilage regeneration and subchondral bone healing.

## 7. Heparin-Based Microspheres and Nanoparticles for Bone Tissue Engineering

Heparin has also been incorporated into nanoparticle- and microsphere-based delivery systems, expanding the design space for controlled release strategies. These nanoscale platforms provide high surface area-to-volume ratios and tunable degradation kinetics, allowing for precise control over the release of growth factors. Importantly, the addition of heparin to these carriers endows them with affinity-based retention, thereby reducing burst release and extending protein bioactivity.

Heparin-conjugated poly(L-lactide-co-glycolide) (PLGA) nanospheres (HCPNs) enabling sustained, near-zero-order release of basic fibroblast growth factor (bFGF, also known as FGF2) were reported by Jeon Q et al. The HCPNs were generated through carbodiimide chemistry, in which heparin was covalently linked to amino-terminated PLGA nanospheres using 1-[3-(dimethylamino)propyl]-3-ethylcarbodiimide. Notably, the heparin conjugation was significantly increased up to 29-fold when nanospheres were fabricated from low-molecular-weight or star-shaped PLGA, as compared to those derived from high-molecular-weight or linear PLGA. Under these conditions, bFGF release from HCPNs could be maintained for approximately three weeks without an initial burst effect. Incorporation of HCPNs into a fibrin gel delivery system further prolonged the release duration to beyond four weeks, while preserving a near-zero-order release profile. The bFGF released from HCPNs embedded in fibrin gel promoted the proliferation of human umbilical vein endothelial cells (HUVECs) for up to 15 days, comparable to cultures receiving daily supplementation with free bFGF. The in vivo results demonstrated that treatment with HCPN-based bFGF delivery significantly increased microvessel density in ischemic limbs more than treatment with daily bFGF injections or bFGF delivery using fibrin gel alone [65].

Chung HJ et al. immobilized heparin on porous PLGA microspheres, which have primary amine groups via covalent conjugation. bFGF was loaded into the heparin-functionalized (PLGA-heparin) microspheres through a simple dipping method. The amount of conjugated amine grouped onto the microspheres was 1.93+/−0.01 nmol/mg-microspheres, while the amount of heparin was 95.8 pmol/mg-microspheres. PLGA–heparin microspheres released bFGF in a more sustained manner with a lesser extent of initial bursting than PLGA microspheres, indicating that surface-immobilized heparin controlled the release rate of bFGF. Subcutaneous implantation of bFGF-loaded PLGA–heparin microspheres in mice significantly induced the formation of new vascular microvessels [66]. Kim SE et al. designed heparin-conjugated PLGA nanoparticles (HCPNs) for sustained release of BMP-2. They found that HCPNs were able to release BMP-2 over a 2-week period. Human BMMSCs cultured in medium containing BMP-2-loaded HCPNs for 2 weeks differentiated toward osteogenic cells, expressed alkaline phosphatase (ALP), osteopontin (OPN), and osteocalcin (OCN) mRNA, while cells without BMP-2 expressed only ALP. In vivo, undifferentiated BMMSCs with BMP-2-loaded HCPNs induced far more extensive bone formation than either implantation of BMP-2-loaded HCPNs or osteogenically differentiated BMMSCs [67].

Tan Q et al. created a heparin/chitosan nanoparticle-immobilized decellularized bovine jugular vein scaffold to increase the loading capacity and allow for controlled release of VEGF. The nanoparticles were then successfully immobilized to the nanofibers of the scaffolds by ethylcarbodiimide hydrochloride/hydroxysulfosuccinimide modification. The scaffolds immobilized with heparin/chitosan nanoparticles exhibited highly effective localization and sustained release of VEGF for several weeks in vitro. This modified scaffold significantly stimulated endothelial cells’ proliferation in vitro. In a mouse subcutaneous implantation model in vivo, the heparin/chitosan nanoparticles used to localize VEGF significantly increased fibroblast infiltration, extracellular matrix production, and accelerated vascularization [68].

Xu X et al. described the fabrication of heparin-functionalized, hyaluronic acid (HA)-based hydrogel particles (HGPs) via an inverse emulsion polymerization approach, using divinyl sulfone as a crosslinking agent. The fabricated microparticles exhibited a spherical morphology with nanoscale porosity, a structural feature that enables growth factor loading. Covalently tethered heparin preserved its specific affinity for BMP-2, and the release behavior of BMP-2 could be finely modulated by adjusting the particle composition. Relative to HA-only particles, hybrid HA/HP HGPs exhibited a markedly enhanced BMP-2 loading capacity. Whereas HA HGPs showed a pronounced initial burst release of BMP-2, HA/HP hybrid particles with an optimized heparin content (0.55 μg/mg HGPs) demonstrated a near-zero-order release profile. Importantly, the controlled release of BMP-2 from HA/HP hybrid particles, together with the intrinsic bioactivity of HA, effectively induced robust chondrogenic differentiation of murine mesenchymal stem cells [69].

Subbiah R et al. evaluated an injectable delivery system composed of heparin microparticles and alginate gel for the dual delivery of VEGF and BMP-2. By modifying the loading strategy, the system enabled either simultaneous or tunable release profiles for both growth factors. In vitro studies demonstrated that the delivery of either VEGF or BMP-2 alone significantly promoted vascularization. However, the combined delivery of VEGF and BMP-2 did not produce a synergistic effect. In a composite bone and muscle injury model, injection of the system resulted in effective bone repair with BMP-2 alone, as well as with both simultaneous and tunable co-delivery of VEGF and BMP-2. Mechanical testing revealed that the tunable delivery of VEGF and BMP-2 restored the strength of the regenerated bone to approximately 52% of that of intact bone, but was insufficient to fully recover bone mechanical properties in this injury model. Considering the severity of the composite injury, VEGF alone appeared inadequate for establishing mature and stable vasculature [70].

Zhou et al. described the development of a heparin-based microsphere–composite hydrogel designed to enable both factor release and stem cell recruitment. The system combined methacrylated gelatin (GelMA), heparin methacrylate (HepMA), and nanohydroxyapatite microspheres loaded with platelet-derived growth factor-BB (PDGF-BB), which were embedded in a GelMA hydrogel containing simvastatin. This dual-release construct provided sustained delivery of both PDGF-BB and simvastatin, effectively inducing rabbit mesenchymal stem cell migration, osteogenic differentiation, and matrix mineralization in vitro, which was confirmed by alkaline phosphatase and alizarin red staining [71].

Taken together, heparin-based nanoparticles and multifunctional delivery systems represent powerful platforms that combine the benefits of nanoscale engineering with affinity-based biochemical interactions. These approaches not only enhance the pharmacokinetic properties of growth factors but also enable the design of controlled release profiles that align with the sequential stages of bone repair, bringing the field closer to clinically viable, low-dose, and effective regenerative therapies. This multiple factor delivery can be used for more complex bone and muscle injury models such as those integrating volumetric muscle loss (VML) with severe segmental bone defects, which is more clinically relevant, especially in traumatic bone and muscle injuries.

**Figure 2:**
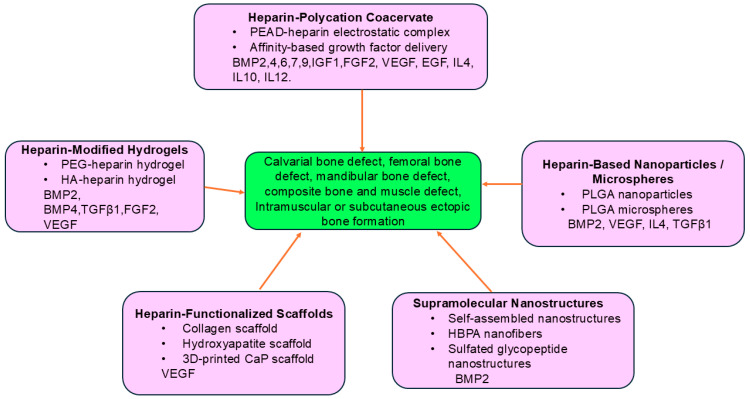
Overview of heparin-based biomaterial platforms for growth factor delivery in bone regeneration. Heparin-based biomaterials can be engineered into multiple delivery platforms, including coacervates, hydrogels, nanoparticles/microspheres, scaffolds, and supramolecular nanostructures. These systems stabilize bioactive molecules and enable controlled release, ultimately promoting bone regeneration.

Taken together, heparin can be used in diversified biomaterials for mediated bone regeneration via its natural growth factor-binding capacity through electrostatic or molecular modification. These applications are summarized in Table 1 below and in Figure 2.

## 8. Clinical Relevance and Translational Outlook

The clinical use of recombinant human bone morphogenetic proteins (rhBMPs) has highlighted both the promise and the risks of growth factor therapy in orthopedics. Randomized and prospective clinical studies support the use of rhBMP-2 for anterior lumbar interbody fusion (ALIF) and for acute open tibial shaft fractures [5,73]. However, widespread use, particularly high-dose delivery on collagen sponges, has revealed important complications including ectopic bone formation, radiculitis, osteolysis, inflammatory swelling, and life-threatening events in cervical applications [6,74,75].

Heparin-based carriers provide sustained, affinity-mediated release that can avoid burst release and significantly lower the dose compared with collagen sponges and can improve spatial localization of BMP-2 in defect regions, thereby reducing ectopic ossification and other side effects. These findings highlight the need for localized, sustained delivery at lower doses. Heparin-based biomaterials may address the current clinical limitations by binding and stabilizing growth factors, extending the half-life of BMP-2 and other growth factors, reducing burst release, and confining activity to the defect site. This localized delivery can lower the risk of ectopic ossification and other off-target effects while reducing the total dose required for efficacy [52]. However, no clinical trials have been reported. Another translational advantage is modularity. Coacervates, hydrogels, and nanoparticle systems can be tailored for single- or multiple-factor delivery, sequential release, or combination with stem cells—an approach aligned with clinical interests in delivering angiogenic, osteogenic, and immunomodulatory cues together [63,71,76]. By lowering the effective dose and minimizing adverse effects, heparin-based delivery systems may broaden growth factor therapies from spinal fusion to indications such as segmental defects, revision arthroplasty, and osteoporotic fractures.

However, further large animal studies and human clinical trials are needed before heparin-based biomaterial delivery systems can be used for human bone tissue repair or regeneration.

## 9. Perspectives

Looking ahead, several key directions are likely to shape the future of heparin-based biomaterials in bone tissue engineering. These include the development of safer derivatives, the design of sequential and multi-factor delivery systems, the integration of cell therapies, translation to large-animal models, and ultimately, clinical trials.

First, although the issue of the anticoagulant activity of heparin is a concern, most current studies on bone tissue engineering focus on localized application and combinations with other hydrogels, scaffolds, or nanoparticles; thus, hemostasis issues have not been reported. While low-anticoagulant derivatives such as ODSH have shown promise in reducing such risks [77], standardized methods are needed to evaluate and report the anticoagulant properties of heparin-based materials [78]. Future research should emphasize chemical modifications that preserve growth factor affinity while eliminating unnecessary systemic effects. Synthetic heparin mimetics and sulfated polymers may offer more reproducible alternatives to animal-derived heparin [79,80].

Second, the design of delivery systems that mimic the spatio-temporal sequence of bone healing will be critical. Natural bone repair involves a process spanning from inflammation to angiogenesis to osteogenesis and remodeling. Heparin-based systems are well suited to staged or sequential delivery, in which angiogenic factors such as VEGF are released first to establish vascular networks, followed by osteoinductive cues like BMP-2 to drive bone formation. Recent advances in hydrogel chemistry and nanoparticle engineering have made it possible to tune release profiles with high precision, enabling biomaterials to orchestrate multiple phases of repair [81,82]. In addition to conventional growth factor delivery, affinity-modified biomaterials have been extended to control the release of bioactive extracellular vesicles. Gao Y et al. demonstrated that heparin-based and polydopamine-functionalized micro-scaffolds could efficiently load and sustain the release of stem cell-derived small extracellular vesicles (sEVs), resulting in enhanced bone regeneration in rat cranial defects [83]. This approach highlights a new dimension of heparin-mediated affinity control that bridges the principles of growth factor delivery with cell-based paracrine signaling, suggesting broader applicability of heparin-engineered systems beyond traditional protein therapeutics.

Third, the integration of heparin-modified scaffolds with stem cell therapies represents a particularly promising avenue. Mesenchymal stem cells (MSCs), endothelial progenitor cells, and induced pluripotent stem cell (iPSC)-derived lineages have been shown to benefit from heparin-containing microenvironments, which improve cell viability, differentiation, and paracrine signaling [84,85,86]. Co-delivery of growth factors and cells within the same construct may further enhance outcomes, especially in large and complex defects where endogenous progenitor recruitment is insufficient [87].

Supporting this concept, Kudaibergen et al. demonstrated that a heparin-conjugated fibrin (HCF) hydrogel co-delivering bone morphogenetic protein-2 (BMP-2) and adipose-derived pericytes significantly enhanced bone regeneration in a rat calvarial critical-sized defect model [72]. The HCF hydrogel provided sustained BMP-2 release, promoted osteogenic differentiation of pericytes in vitro, and achieved superior defect closure in vivo compared to single-component treatments. These findings highlight the synergistic potential of combining heparin-based growth factor delivery with cell transplantation for large-defect repair.

Fourth, translation will depend on rigorous evaluation in large-animal models that better replicate human bone healing. While rodent models have been indispensable for proof-of-concept studies, differences in defect size, loading environment, and immune response necessitate testing in clinically relevant species such as sheep, goats, and non-human primates [88]. Advances in GMP-compatible synthesis of heparin derivatives and scalable fabrication of hydrogels, scaffolds, and nanoparticles will accelerate clinical translation.

In summary, the advancement of heparin-based biomaterials will depend on their capacity to enable physiological levels of multiple growth factors, their integration with cell-based therapies, and their translation toward clinical use through validation in large-animal models and regulatory readiness. By addressing safety, standardization, and scalability, these systems have the potential to transform bone tissue engineering and provide reliable, low-dose, and patient-specific regenerative solutions.

## 10. Conclusions

In summary, heparin can be used in a variety of biomaterial platforms for the controlled delivery of growth factors and promoting bone regeneration. Heparin–PEAD coacervate has been widely used for bone tissue engineering and has been used for the sustained release of BMP-2, -4, -6, -7, and -9 and IGF1 for bone tissue engineering using both critical-sized calvarial bone defects and ectopic bone regeneration models. Heparin–PEAD has also been used for the sustained release of BMP-2, -4, -6, -7, and -9 for microfracture osteochondral defect repair and can deliver BMP-2 and sFLT1 simultaneously for MIA-mediated osteoarthritis repair or in combination with ADSCs and IGF1 to promote osteochondral repair. The heparin–PEAD coacervate sustained release platform does not incur any adverse effects due to its anti-coagulating effect. It can even be used with fibrin sealant (Tisseel, Baxter, FDA approved) without affecting fibrin gel formation for bone, cartilage, and soft tissues such as the infarcted heart [24,29,40,89,90,91]. It is injectable and suitable for not only bone tissue engineering but also osteoarthritis and regeneration of other soft tissues and can be completely absorbed after 6 weeks [25]. Therefore, it is safe.

The heparin-based PA-nanofiber supramolecule has also been shown to significantly reduce the BMP-2 dose and HO formation in long bone defects and spine fusion models. Heparin-based hydrogels represent one of the most widely investigated delivery platforms because their highly hydrated networks help preserve protein bioactivity while enabling sustained release of therapeutic factors. These materials can also be injected to fit irregular defect geometries, making them particularly attractive for minimally invasive regenerative strategies. Heparin-based porous scaffolds provide greater structural stability and mechanical support, which can be advantageous in large bone defects or load-bearing applications. Heparin-based microspheres and nanoparticles have also been developed for promoting bone regeneration, including in composite bone and muscle injury models. However, each platform also presents limitations, including differences in mechanical properties, release profiles, and translational feasibility. Therefore, selecting an appropriate delivery system requires careful consideration of the clinical context and therapeutic goals [92].

Furthermore, heparin can bind up to 300 growth factors. Several growth factors have been extensively investigated in combination with heparin-based delivery systems for bone regeneration. Bone morphogenetic proteins (BMP-2, -4, -6, -7, and -9), particularly BMP-2 and BMP-7, are among the most frequently studied because of their strong osteoinductive properties. Heparin can bind to these proteins and stabilize them within biomaterial matrices, achieving sustained release, thereby prolonging their biological activity and improving therapeutic efficiency. VEGF is also commonly incorporated to stimulate angiogenesis, an essential process for successful bone repair. The combination of fibroblast growth factor-2 (FGF-2) and platelet-derived growth factors (PDGFs), particularly when their spatial presentation is controlled, is highly effective and particularly suitable for combinatorial regenerative strategies [93].

Despite the promising progress of heparin-based biomaterials for regenerative medicine, several important challenges remain for successful clinical translation. Pharmaceutical heparin is typically derived from animal tissues, which may introduce batch-to-batch variability and potential contamination risks during large-scale production. In addition, although modified heparins with reduced anticoagulant activity have been developed, safety considerations such as bleeding risk and potential immunogenic responses must still be carefully evaluated. Regulatory approval may also be complex, as many heparin-containing biomaterials are classified as drug–device combination products. Furthermore, issues related to reproducibility, scalability, and manufacturing cost remain important considerations for the future clinical application of these technologies [93].

Looking ahead, challenges remain in balancing growth factor affinity with anticoagulant activity, developing reproducible synthetic mimetics, and scaling up manufacturing to GMP standards. Nonetheless, the modularity and translational feasibility of heparin-based systems and further combined evidence from large-animal models will provide a clear path toward clinical application.

In summary, heparin-based biomaterials are poised to become key enablers in regenerative medicine, offering precise, durable, and biologically responsive delivery platforms that bridge fundamental science and translational therapy.

## Figures and Tables

**Table 1:** Summary of heparin-based biomaterial systems used for controlled growth factor delivery in bone tissue engineering.

Heparin-Based Biomaterial System	Growth Factor(s)	Delivery Strategy (Scaffold)	Experimental Model	Key Outcome	Authors and Reference
Heparin–PEAD coacervate-based sustained release	BMP-2	Heparin–polycation electrostatic complex enabling sustained release with fibrin sealant scaffold	Ectopic bone formation	Sustained BMP-2 delivery promotes osteogenesis and bone regeneration	Li, H. et al. [33]
Heparin–PEAD coacervate-based sustained release	BMP-2,4,6,7,9	Heparin–polycation electrostatic complex enabling sustained release of combined fibrin sealant scaffold	Mouse critical-sized calvarial bone defect	Coacervate-based sustained release of BMPs promotes bone regeneration, with BMP-2 and 7 being the most potent	Gao X et al. [25]
Heparin–PEAD coacervate-based sustained release	BMP-2	Heparin–polycation electrostatic complex enabling sustained release of combined thiolated gelatin/poly(ethylene glycol) diacrylate (PEGDA) microparticle hydrogel	Rat calvarial bone defect	Increased bone formation area and bone bridging	Kim S et al. [34]
Heparin–PEAD coacervate-based sustained release	BMP-2 and sFLT1	Heparin–polycation electrostatic complex enabling sustained release without scaffold	Nude rat MIA-induced osteoarthritis	Promoted cartilage repair, similar to lenti-BMP-2-mediated gene therapy	Gao et al. [37]
Heparin–PEAD coacervate-based sustained release	IGF1 and ADSCs	Heparin–polycation electrostatic complex enabling sustained release combined with Gelatin-SH/PEGDA IPN hydrogels	Rabbit osteochondral defect	Enhanced cartilage and subchondral bone healing	Cho, H et al. [41]
Heparin–PEAD coacervate-based sustained release	BMP-2,4,6,7,9	Heparin–polycation electrostatic complex enabling sustained release with fibrin sealant scaffold	Rat trochlear groove osteochondral defect	Enhanced microfracture-mediated cartilage repair via promoting subchondral bone healing and up-regulating SOX9	Gao X et al. [26]
Heparin-based supramolecules	BMP-2	Heparin-binding peptide amphiphile (HBPA) nanofibers with syndecan and fibronectin allow sustained release	Rat 5 mm critical-sized femoral defect	More effective in bone bridging and more mature bone than PA nanofiber alone	Lee S et al. [42]
Heparin-based supramolecules	BMP-2	Heparin mimetric with thiosulphate monosaccharide-enhanced growth factor binding that can bind five different proteins with heparin domain	Spine fusion model	100 ng BMP-2 induced complete spine fusion	Lee S et al. [43]
Heparin hydrogel	FGF2 and VEGF	Heparin-bound growth factor combined with StarPEG hydrogels	In vitro	Promotes differentiation of HUVECs	Zieris A et al. [46]
Heparin hydrogel	BMP-2	Heparin-bound growth factor-based sustained release; conjugated with fibrin	Mouse calvarial bone defect	Complete healing of calvarial bone defect in 8 weeks using 0.5 BMP-2 delivered with collagen sponge (2 μg)	Yang HS et al. [47]
Heparin hydrogel	BMP-2	Heparin sulfate-bound growth factor conjugated with Carboxymethyl chitosan (CMCS)/polyethylene glycol (PEG) (CMCS/PEG/HS) formed hydrogel	Rat 3 mm calvarial bone defect model	CMCS/PEG/HS loaded with 0.5 μg BMP-2 nearly completely healed the defect	Zhou T et al. [49]
Heparin hydrogel	BMP-2	Gelatin–heparin–tyramine hydrogel for sustained release of growth factor	Idiopathic osteonecrosis of the femoral head	Produced homogeneous bone regeneration without HO; preserved the subchondral contour and restored subchondral endochondral ossification	Ma C et al. [50]
Heparin hydrogel	BMP2	Heparin-bound growth factor conjugated with Hyaluronan-based hydrogels (Heprasil™) for sustained release	Rat muscle ectopic bone formation model	Sustained BMP-2 release and enhanced bone formation	Bhakta et al. [48]
Heparin hydrogel	BMP-2	Heparin-bound growth factor in combination with gelatin/polyethylene glycol diacrylate/2- (dimethylamino)ethyl methacrylate, GPEGD) to form hydrogel for sustained release	Rat mandibular 5 mm bone defect	Promoted bone formation via osteoinduction and anti-oxidation	Wu Y et al. [51]
Heparin hydrogel	BMP-2	Heparin microparticle (HMP)-bound growth factor incorporated into alginate hydrogel surrounded by a perforated poly(caprolactone) (PCL) nanofiber mesh for sustained release	Rat femoral bone defect	Increased BMP-2 retention in defect site; reduced heterotopic bone formation	Hettiaratchi MH et al. [52]
Heparin nanomembrane hydrogel	VEGF, BMP-2, NGF	Heparin-modified electrospun gelatin/polycaprolactone (GEL/PCL) nanofiber membranes for sustained, controlled release	Subcutaneous semiorthotopic transplantation model	Facilitated vascular and neural ingrowth of dental pulp-like tissue and odontogen formation	Li X et al. [54]
Heparin hydrogel	FGF	Heparan sulfate proteoglycan 2(HSPG2)-coated poly (lactic acid) (PLA) for sustained release	Cranial defect	Creation of an osteoconductive environment; repaired cranial bone defects	Ku CY et al. [56]
Heparin hydrogel	BMP-2	Heparin-bound growth factor crosslinked with Tilapia skin collagen using EDC/NHS	Rat calvarial bone defect	Increased bone volume and BV density compared to Collagen-BMP-2	Ma L et al. [57]
Heparin hydrogel	TGFβ1 and BMP4 with SDMSCs	Heparin-conjugated fibrin (HCF) hydrogel bound to growth factor for sustained release	Rabbit osteochondral defect	Enhanced regeneration of hyaline cartilage and subchondral bone plate with osteochondral defects	Sarsenova, M et al. [63]
Heparin nanogel	BMP-2	Heparin-bound BMP2 conjugated with PEI to form nanogels (NP/BMP2) and immobilized onto BCP substrates scaffolds, allowing for sustained release	Rat femoral condyle defect and intra-muscular implantation	Promoted bone repair and formation	Tang Y et al. [59]
Heparin hydrogel	BMP-2 and pericytes	Heparin-bound growth factor-conjugated fibrin (HCF) hydrogel for sustained release	Rat critical-sized calvarial bone defect model	HCF co-delivery of BMP-2 and pericytes enhanced bone regeneration more than HCF-BMP-2 or HCF-pericytes	Kudaibergen G et al. [72]
Heparinized inorganic porous scaffold	VEGF	Heparinized bound growth factor in conjugated gelatin–hydroxyapatite–TCP scaffold for sustained release	Rat calvarial defect model	Enhanced angiogenesis and bone regeneration	Chen X et al. [60]
Heparin-modified mineralized collagen	BMP-2 and natural bimixture	Heparin-modified mineralized collagen scaffolds for sustained release of natural growth factor	Mouse 2 mm segmental femoral defect	Promoted new bone formation and defect healing	Vater C et al. [61]
Heparin-conjugated microparticle	IL4 and TGFβ1	Heparin-bound growth factor conjugated with Methacrylated hyaluronic acid (HAMA@HepMA) to form microparticles for sustained release	Rat trochlear groove osteochondral defect	Decreased inflammation, enhanced cartilage repair, and subchondral bone healing	Lin C et al. [64]
Heparin nanosphere	BMP-2 and MSCSs	Heparin-conjugated poly(L-lactide-co-glycolide) (PLGA) nanospheres (HCPNs) embedded in fibrin gel scaffold for sustained release	Mouse subcutaneous ectopic bone formation	More extensive bone formation than HCPNs with BMP-2	Kim S et al. [67]
Heparin nanoparticle	VEGF	Heparin/chitosan nanoparticle-immobilized decellularized bovine jugular vein scaffold for sustainedrelease	Mouse subcutaneous implantation model in vivo	Accelerated vas cularization	Tan Q et al. [68]
Heparin-conjugated nanoparticle	BMP-2	Heparin (HP)-bound growth factor in conjugation with hyaluronic acid (HA) to form hydrogel particles (HGPs) for sustained release	In vitro	Sustained release and promoted chondrogenic differentiation	Xu X et al. [69]
Heparin microparticle	BMP-2 and VEGF	Heparin microparticle-bound growth factor and combined with alginate gel for sustained release	Composite femoral segmental bone defect and volumetric muscle loss	Effective bone healing was achieved in all treatment groups (simultaneous or tunable delivery of BMP-2 and VEGF)	Subbiah R et al. [70]
Heparin microspheres	bFGF	Heparin-bound growth factor immobilized with PLGA microspheres for sustained growth factor delivery	Mouse subcutaneous implantation model	Sustained release with reduced bursting and enhanced angiogenesis	Chung H J et al. [66]

## Data Availability

Not applicable as this is a review article.

## References

[1] McKibbin B. (1978). The biology of fracture healing in long bones. J. Bone Jt. Surg. Br..

[2] Gerstenfeld L.C., Cullinane D.M., Barnes G.L., Graves D.T., Einhorn T.A. (2003). Fracture healing as a post-natal developmental process: Molecular, spatial, and temporal aspects of its regulation. J. Cell Biochem..

[3] Marsell R., Einhorn T.A. (2011). The biology of fracture healing. Injury.

[4] Bauer T.W., Muschler G.F. (2000). Bone graft materials. An overview of the basic science. Clin. Orthop. Relat. Res..

[5] Govender S., Csimma C., Genant H.K., Valentin-Opran A., Amit Y., Arbel R., Aro H., Atar D., Bishay M., Borner M.G. (2002). Recombinant human bone morphogenetic protein-2 for treatment of open tibial fractures: A prospective, controlled, randomized study of four hundred and fifty patients. J. Bone Jt. Surg. Am..

[6] Carragee E.J., Hurwitz E.L., Weiner B.K. (2011). A critical review of recombinant human bone morphogenetic protein-2 trials in spinal surgery: Emerging safety concerns and lessons learned. Spine J..

[7] Simmonds M.C., Brown J.V., Heirs M.K., Higgins J.P., Mannion R.J., Rodgers M.A., Stewart L.A. (2013). Safety and effectiveness of recombinant human bone morphogenetic protein-2 for spinal fusion: A meta-analysis of individual-participant data. Ann. Intern. Med..

[8] Wang J.F., Jan J.S., Hu J.J. (2025). Heparin-Based Growth Factor Delivery Platforms: A Review. Pharmaceutics.

[9] Gatica Portillo D.R., Li Y., Goyal N., Rowan B.G., Al-Horani R.A., Anbalagan M. (2025). Heparin, Heparin-like Molecules, and Heparin Mimetics in Breast Cancer: A Concise Review. Biomolecules.

[10] Rudd T.R., Preston M.D., Yates E.A. (2017). The nature of the conserved basic amino acid sequences found among 437 heparin binding proteins determined by network analysis. Mol. Biosyst..

[11] Chiodelli P., Bugatti A., Urbinati C., Rusnati M. (2015). Heparin/Heparan sulfate proteoglycans glycomic interactome in angiogenesis: Biological implications and therapeutical use. Molecules.

[12] Capila I., Linhardt R.J. (2002). Heparin-protein interactions. Angew. Chem. Int. Ed. Engl..

[13] Xu D., Esko J.D. (2014). Demystifying heparan sulfate-protein interactions. Annu. Rev. Biochem..

[14] Ori A., Wilkinson M.C., Fernig D.G. (2011). A systems biology approach for the investigation of the heparin/heparan sulfate interactome. J. Biol. Chem..

[15] Johnson N.R., Ambe T., Wang Y. (2014). Lysine-based polycation:heparin coacervate for controlled protein delivery. Acta Biomater..

[16] Carlsson P., Kjellen L. (2012). Heparin Biosynthesis.

[17] Powell A.K., Yates E.A., Fernig D.G., Turnbull J.E. (2004). Interactions of heparin/heparan sulfate with proteins: Appraisal of structural factors and experimental approaches. Glycobiology.

[18] Meneghetti M.C., Hughes A.J., Rudd T.R., Nader H.B., Powell A.K., Yates E.A., Lima M.A. (2015). Heparan sulfate and heparin interactions with proteins. J. R. Soc. Interface.

[19] Sarrazin S., Lamanna W.C., Esko J.D. (2011). Heparan sulfate proteoglycans. Cold Spring Harb. Perspect. Biol..

[20] Ruppert R., Hoffmann E., Sebald W. (1996). Human bone morphogenetic protein 2 contains a heparin-binding site which modifies its biological activity. Eur. J. Biochem..

[21] Ornitz D.M., Itoh N. (2015). The Fibroblast Growth Factor signaling pathway. Wiley Interdiscip. Rev. Dev. Biol..

[22] Martino M.M., Tortelli F., Mochizuki M., Traub S., Ben-David D., Kuhn G.A., Muller R., Livne E., Eming S.A., Hubbell J.A. (2011). Engineering the growth factor microenvironment with fibronectin domains to promote wound and bone tissue healing. Sci. Transl. Med..

[23] Sakiyama-Elbert S.E. (2014). Incorporation of heparin into biomaterials. Acta Biomater..

[24] Chu H., Chen C.W., Huard J., Wang Y. (2013). The effect of a heparin-based coacervate of fibroblast growth factor-2 on scarring in the infarcted myocardium. Biomaterials.

[25] Gao X., Hwang M.P., Wright N., Lu A., Ruzbarsky J.J., Huard M., Cheng H., Mullen M., Ravuri S., Wang B. (2022). The use of heparin/polycation coacervate sustain release system to compare the bone regenerative potentials of 5 BMPs using a critical sized calvarial bone defect model. Biomaterials.

[26] Gao X., Wright N., Huard M., Tan J., Ruzbarsky J., Lu A., Chubb L., Tuan R., Philippon M.J., Wang Y. (2025). Comparison of 5 BMPs for their chondrogenic potentials and microfracture-mediated cartilage repair using heparin/PEAD coacervate sustained release polymer. Bioact. Mater..

[27] Cassinelli G., Naggi A. (2016). Old and new applications of non-anticoagulant heparin. Int. J. Cardiol..

[28] Rao N.V., Argyle B., Xu X., Reynolds P.R., Walenga J.M., Prechel M., Prestwich G.D., MacArthur R.B., Walters B.B., Hoidal J.R. (2010). Low anticoagulant heparin targets multiple sites of inflammation, suppresses heparin-induced thrombocytopenia, and inhibits interaction of RAGE with its ligands. Am. J. Physiol. Cell Physiol..

[29] Chu H., Gao J., Chen C.W., Huard J., Wang Y. (2011). Injectable fibroblast growth factor-2 coacervate for persistent angiogenesis. Proc. Natl. Acad. Sci. USA.

[30] Chu H., Gao J., Wang Y. (2012). Design, synthesis, and biocompatibility of an arginine-based polyester. Biotechnol. Prog..

[31] Xie Y., Upton Z., Richards S., Rizzi S.C., Leavesley D.I. (2011). Hyaluronic acid: Evaluation as a potential delivery vehicle for vitronectin:growth factor complexes in wound healing applications. J. Control Release.

[32] Chu H., Johnson N.R., Mason N.S., Wang Y. (2011). A [polycation:heparin] complex releases growth factors with enhanced bioactivity. J. Control Release.

[33] Li H., Johnson N.R., Usas A., Lu A., Poddar M., Wang Y., Huard J. (2013). Sustained release of bone morphogenetic protein 2 via coacervate improves the osteogenic potential of muscle-derived stem cells. Stem Cells Transl. Med..

[34] Kim S., Kim J., Gajendiran M., Yoon M., Hwang M.P., Wang Y., Kang B.J., Kim K. (2018). Enhanced Skull Bone Regeneration by Sustained Release of BMP-2 in Interpenetrating Composite Hydrogels. Biomacromolecules.

[35] Siverino C., Fahmy-Garcia S., Niklaus V., Kops N., Dolcini L., Misciagna M.M., Ridwan Y., Farrell E., van Osch G., Nickel J. (2023). Addition of heparin binding sites strongly increases the bone forming capabilities of BMP9 in vivo. Bioact. Mater..

[36] Patel Z.S., Young S., Tabata Y., Jansen J.A., Wong M.E., Mikos A.G. (2008). Dual delivery of an angiogenic and an osteogenic growth factor for bone regeneration in a critical size defect model. Bone.

[37] Gao X., Cheng H., Awada H., Tang Y., Amra S., Lu A., Sun X., Lv G., Huard C., Wang B. (2019). A comparison of BMP2 delivery by coacervate and gene therapy for promoting human muscle-derived stem cell-mediated articular cartilage repair. Stem Cell Res. Ther..

[38] Lee M.S., Ahmad T., Lee J., Awada H.K., Wang Y., Kim K., Shin H., Yang H.S. (2017). Dual delivery of growth factors with coacervate-coated poly(lactic-co-glycolic acid) nanofiber improves neovascularization in a mouse skin flap model. Biomaterials.

[39] Park U., Lee M.S., Jeon J., Lee S., Hwang M.P., Wang Y., Yang H.S., Kim K. (2019). Coacervate-mediated exogenous growth factor delivery for scarless skin regeneration. Acta Biomater..

[40] Chen W.C., Lee B.G., Park D.W., Kim K., Chu H., Kim K., Huard J., Wang Y. (2015). Controlled dual delivery of fibroblast growth factor-2 and Interleukin-10 by heparin-based coacervate synergistically enhances ischemic heart repair. Biomaterials.

[41] Cho H., Kim J., Kim S., Jung Y.C., Wang Y., Kang B.J., Kim K. (2020). Dual delivery of stem cells and insulin-like growth factor-1 in coacervate-embedded composite hydrogels for enhanced cartilage regeneration in osteochondral defects. J. Control Release.

[42] Lee S.S., Huang B.J., Kaltz S.R., Sur S., Newcomb C.J., Stock S.R., Shah R.N., Stupp S.I. (2013). Bone regeneration with low dose BMP-2 amplified by biomimetic supramolecular nanofibers within collagen scaffolds. Biomaterials.

[43] Lee S.S., Fyrner T., Chen F., Alvarez Z., Sleep E., Chun D.S., Weiner J.A., Cook R.W., Freshman R.D., Schallmo M.S. (2017). Sulfated glycopeptide nanostructures for multipotent protein activation. Nat. Nanotechnol..

[44] McClendon M.T., Ji W., Greene A.C., Sai H., Sangji M.H., Sather N.A., Chen C.H., Lee S.S., Katchko K., Jeong S.S. (2023). A supramolecular polymer-collagen microparticle slurry for bone regeneration with minimal growth factor. Biomaterials.

[45] Newcomb C.J., Sur S., Lee S.S., Yu J.M., Zhou Y., Snead M.L., Stupp S.I. (2016). Supramolecular Nanofibers Enhance Growth Factor Signaling by Increasing Lipid Raft Mobility. Nano Lett..

[46] Zieris A., Prokoph S., Levental K.R., Welzel P.B., Grimmer M., Freudenberg U., Werner C. (2010). FGF-2 and VEGF functionalization of starPEG-heparin hydrogels to modulate biomolecular and physical cues of angiogenesis. Biomaterials.

[47] Yang H.S., La W.G., Cho Y.M., Shin W., Yeo G.D., Kim B.S. (2012). Comparison between heparin-conjugated fibrin and collagen sponge as bone morphogenetic protein-2 carriers for bone regeneration. Exp. Mol. Med..

[48] Bhakta G., Rai B., Lim Z.X., Hui J.H., Stein G.S., van Wijnen A.J., Nurcombe V., Prestwich G.D., Cool S.M. (2012). Hyaluronic acid-based hydrogels functionalized with heparin that support controlled release of bioactive BMP-2. Biomaterials.

[49] Zhou T., Wang F., Liu K., Zhou H., Shang J. (2024). An injectable carboxymethyl chitosan-based hydrogel with controlled release of BMP-2 for efficient treatment of bone defects. Int. J. Biol. Macromol..

[50] Ma C., Park M.S., Alves do Monte F., Gokani V., Aruwajoye O.O., Ren Y., Liu X., Kim H.K.W. (2023). Local BMP2 hydrogel therapy for robust bone regeneration in a porcine model of Legg-Calve-Perthes disease. NPJ Regen. Med..

[51] Wu Y., Li X., Sun Y., Tan X., Wang C., Wang Z., Ye L. (2023). Multiscale design of stiffening and ROS scavenging hydrogels for the augmentation of mandibular bone regeneration. Bioact. Mater..

[52] Hettiaratchi M.H., Krishnan L., Rouse T., Chou C., McDevitt T.C., Guldberg R.E. (2020). Heparin-mediated delivery of bone morphogenetic protein-2 improves spatial localization of bone regeneration. Sci. Adv..

[53] Brown N.E., Ellerbe L.R., Hollister S.J., Temenoff J.S. (2024). Development and Characterization of Heparin-Containing Hydrogel/3D-Printed Scaffold Composites for Craniofacial Reconstruction. Ann. Biomed. Eng..

[54] Li X.L., Liu H., Fan W., Fan B. (2025). An Electrospun Heparin Modified Nanofiber Membrane Carrying Multiple Growth Factors for Dental Pulp Regeneration. Int. Dent. J..

[55] Ishihara J., Ishihara A., Fukunaga K., Sasaki K., White M.J.V., Briquez P.S., Hubbell J.A. (2018). Laminin heparin-binding peptides bind to several growth factors and enhance diabetic wound healing. Nat. Commun..

[56] Ku C.Y., Chen Y.H., Lin C.M., Chu Y.H., Ho Y.J., Li-Ling L., Huang Y.C., Okuyama K., Liu C.H., Liao W.C. (2026). Enhancing the efficiency of bone tissue regeneration by using a 3D printed scaffold optimized with heparan sulfate proteoglycan 2. Biomed. Mater..

[57] Ma L., Fu L., Gu C., Wang H., Yu Z., Gao X., Zhao D., Ge B., Zhang N. (2023). Delivery of bone morphogenetic protein-2 by crosslinking heparin to nile tilapia skin collagen for promotion of rat calvaria bone defect repair. Prog. Biomater..

[58] Jiang M., Pan Y., Liu Y., Dai K., Zhang Q., Wang J. (2022). Effect of sulfated chitosan hydrogel on vascularization and osteogenesis. Carbohydr. Polym..

[59] Tang Y., Wang J., Cao Q., Chen F., Wang M., Wu Y., Chen X., Zhu X., Zhang X. (2022). Dopamine/DOPAC-assisted immobilization of bone morphogenetic protein-2 loaded Heparin/PEI nanogels onto three-dimentional printed calcium phosphate ceramics for enhanced osteoinductivity and osteogenicity. Biomater. Adv..

[60] Chen X., Gao C.Y., Chu X.Y., Zheng C.Y., Luan Y.Y., He X., Yang K., Zhang D.L. (2022). VEGF-Loaded Heparinised Gelatine-Hydroxyapatite-Tricalcium Phosphate Scaffold Accelerates Bone Regeneration via Enhancing Osteogenesis-Angiogenesis Coupling. Front. Bioeng. Biotechnol..

[61] Vater C., Hetz M., Quade M., Lode A., Gelinsky M., Rammelt S., Zwingenberger S., Bretschneider H. (2023). Combined application of BMP-2 and naturally occurring bioactive factor mixtures for the optimized therapy of segmental bone defects. Acta Biomater..

[62] Sun X., Wang J., Wang Y., Huang C., Yang C., Chen M., Chen L., Zhang Q. (2018). Scaffold with Orientated Microtubule Structure Containing Polylysine-Heparin Sodium Nanoparticles for the Controlled Release of TGF-beta1 in Cartilage Tissue Engineering. ACS Appl. Bio Mater..

[63] Sarsenova M., Raimagambetov Y., Issabekova A., Karzhauov M., Kudaibergen G., Akhmetkarimova Z., Batpen A., Ramankulov Y., Ogay V. (2022). Regeneration of Osteochondral Defects by Combined Delivery of Synovium-Derived Mesenchymal Stem Cells, TGF-beta1 and BMP-4 in Heparin-Conjugated Fibrin Hydrogel. Polymers.

[64] Lin C., Ying C., Xu Y., Zou Y., Chen R., Xu K., Ji X., Cao Q., Weng J., Jiang L. (2025). Synergizing adaptive immunity and regenerative signals to enhance osteochondral defects repair. Bioact. Mater..

[65] Jeon O., Kang S.W., Lim H.W., Hyung Chung J., Kim B.S. (2006). Long-term and zero-order release of basic fibroblast growth factor from heparin-conjugated poly(L-lactide-co-glycolide) nanospheres and fibrin gel. Biomaterials.

[66] Chung H.J., Kim H.K., Yoon J.J., Park T.G. (2006). Heparin immobilized porous PLGA microspheres for angiogenic growth factor delivery. Pharm. Res..

[67] Kim S.E., Jeon O., Lee J.B., Bae M.S., Chun H.J., Moon S.H., Kwon I.K. (2008). Enhancement of ectopic bone formation by bone morphogenetic protein-2 delivery using heparin-conjugated PLGA nanoparticles with transplantation of bone marrow-derived mesenchymal stem cells. J. Biomed. Sci..

[68] Tan Q., Tang H., Hu J., Hu Y., Zhou X., Tao Y., Wu Z. (2011). Controlled release of chitosan/heparin nanoparticle-delivered VEGF enhances regeneration of decellularized tissue-engineered scaffolds. Int. J. Nanomed..

[69] Xu X., Jha A.K., Duncan R.L., Jia X. (2011). Heparin-decorated, hyaluronic acid-based hydrogel particles for the controlled release of bone morphogenetic protein 2. Acta Biomater..

[70] Subbiah R., Cheng A., Ruehle M.A., Hettiaratchi M.H., Bertassoni L.E., Guldberg R.E. (2020). Effects of controlled dual growth factor delivery on bone regeneration following composite bone-muscle injury. Acta Biomater..

[71] Zhou J., Zhao Y., Ling Y., Zhao P., Gao H., Yang Y., Chen J. (2024). Microsphere-Composite Hydrogel for Recruiting Stem Cells and Promoting Osteogenic Differentiation. ACS Appl. Bio Mater..

[72] Kudaibergen G., Mukhlis S., Mukhambetova A., Issabekova A., Sekenova A., Sarsenova M., Temirzhan A., Baidarbekov M., Umbayev B., Ogay V. (2024). Repair of Rat Calvarial Critical-Sized Defects Using Heparin-Conjugated Fibrin Hydrogel Containing BMP-2 and Adipose-Derived Pericytes. Bioengineering.

[73] Burkus J.K., Transfeldt E.E., Kitchel S.H., Watkins R.G., Balderston R.A. (2002). Clinical and radiographic outcomes of anterior lumbar interbody fusion using recombinant human bone morphogenetic protein-2. Spine.

[74] Perri B., Cooper M., Lauryssen C., Anand N. (2007). Adverse swelling associated with use of rh-BMP-2 in anterior cervical discectomy and fusion: A case study. Spine J..

[75] Villavicencio A.T., Burneikiene S. (2016). RhBMP-2-induced radiculitis in patients undergoing transforaminal lumbar interbody fusion: Relationship to dose. Spine J..

[76] Johnson N.R., Wang Y. (2014). Coacervate delivery systems for proteins and small molecule drugs. Expert. Opin. Drug Deliv..

[77] Tkaczynski E., Arulselvan A., Tkaczynski J., Avery S., Xiao L., Torok-Storb B., Abrams K., Rao N.V., Johnson G., Kennedy T.P. (2018). 2-O, 3-O desulfated heparin mitigates murine chemotherapy- and radiation-induced thrombocytopenia. Blood Adv..

[78] Gray E., Mulloy B., Barrowcliffe T.W. (2008). Heparin and low-molecular-weight heparin. Thromb. Haemost..

[79] Petitou M., van Boeckel C.A. (2004). A synthetic antithrombin III binding pentasaccharide is now a drug! What comes next?. Angew. Chem. Int. Ed. Engl..

[80] Li J.P., Kusche-Gullberg M. (2016). Heparan Sulfate: Biosynthesis, Structure, and Function. Int. Rev. Cell Mol. Biol..

[81] Ma P.X. (2008). Biomimetic materials for tissue engineering. Adv. Drug Deliv. Rev..

[82] Wei Z., Volkova E., Blatchley M.R., Gerecht S. (2019). Hydrogel vehicles for sequential delivery of protein drugs to promote vascular regeneration. Adv. Drug Deliv. Rev..

[83] Gao Y., Yuan X., Gu R., Wang N., Ren H., Song R., Wan Z., Huang J., Yi K., Xiong C. (2025). Affinity Modifications of Porous Microscaffolds Impact Bone Regeneration by Modulating the Delivery Kinetics of Small Extracellular Vesicles. ACS Nano.

[84] Benoit D.S., Durney A.R., Anseth K.S. (2007). The effect of heparin-functionalized PEG hydrogels on three-dimensional human mesenchymal stem cell osteogenic differentiation. Biomaterials.

[85] Yang H.N., Choi J.H., Park J.S., Jeon S.Y., Park K.D., Park K.H. (2014). Differentiation of endothelial progenitor cells into endothelial cells by heparin-modified supramolecular pluronic nanogels encapsulating bFGF and complexed with VEGF165 genes. Biomaterials.

[86] Arkenberg M.R., Koehler K., Lin C.C. (2022). Heparinized Gelatin-Based Hydrogels for Differentiation of Induced Pluripotent Stem Cells. Biomacromolecules.

[87] Vo T.N., Kasper F.K., Mikos A.G. (2012). Strategies for controlled delivery of growth factors and cells for bone regeneration. Adv. Drug Deliv. Rev..

[88] Pearce A.I., Richards R.G., Milz S., Schneider E., Pearce S.G. (2007). Animal models for implant biomaterial research in bone: A review. Eur. Cell Mater..

[89] Wang Z., Long D.W., Huang Y., Khor S., Li X., Jian X., Wang Y. (2017). Fibroblast Growth Factor-1 Released from a Heparin Coacervate Improves Cardiac Function in a Mouse Myocardial Infarction Model. ACS Biomater. Sci. Eng..

[90] Johnson N.R., Kruger M., Goetsch K.P., Zilla P., Bezuidenhout D., Wang Y., Davies N.H. (2015). Coacervate Delivery of Growth Factors Combined with a Degradable Hydrogel Preserves Heart Function after Myocardial Infarction. ACS Biomater. Sci. Eng..

[91] Awada H.K., Johnson N.R., Wang Y. (2015). Sequential delivery of angiogenic growth factors improves revascularization and heart function after myocardial infarction. J. Control Release.

[92] Tang X., Zhou F., Wang S., Wang G., Bai L., Su J. (2025). Bioinspired injectable hydrogels for bone regeneration. J. Adv. Res..

[93] Takematsu E., Murphy M., Hou S., Steininger H., Alam A., Ambrosi T.H., Chan C.K.F. (2023). Optimizing Delivery of Therapeutic Growth Factors for Bone and Cartilage Regeneration. Gels.

